# Avoidable delays in the care of infective endocarditis patients: a priority issue

**DOI:** 10.3389/fcvm.2026.1847517

**Published:** 2026-08-03

**Authors:** Pasquale Baratta, Alberto Cresti, Francesco De Sensi, Bruno Sposato, Andrea Picchi, Ugo Limbruno

**Affiliations:** 1 Cardiology Department, Misericordia Hospital, Grosseto, Italy; 2 Internal Medicine Department, Misericordia Hospital, Grosseto, Italy

**Keywords:** diagnostic delay, endocarditis, Heart Team, mortality, surgery delay

## Abstract

**Background:**

Guidelines recommend prompt diagnosis and timely surgical intervention for infective endocarditis (IE); however, adherence in clinical practice remains inconsistent. We quantified delays throughout the IE care pathway and evaluated their association with mortality after adjusting for baseline severity.

**Methods:**

We conducted a retrospective single-centre cohort study of consecutive patients with definite IE and a guideline-based surgical indication who were admitted to Misericordia Hospital, Grosseto (Italy), between 1 January 1998 and 31 December 2023. Time intervals from the first symptom to hospital admission, to transthoracic (TTE), transoesophageal (TOE) echocardiography, and surgery were recorded. Patients were classified as In-Time if surgery was performed within the guideline-recommended timeframe (emergency: <24 h; urgent: <5 days; elective: otherwise) or Delayed. A prespecified subgroup analysis within the Delayed group distinguished Delayed-operated from Delayed-never-operated patients. Univariable and multivariable logistic regression estimated the association between delay and in-hospital mortality, adjusting for age, Sequential Organ Failure Assessment (SOFA) score, *Staphylococcus aureus* aetiology, acute heart failure, embolization, and abscess.

**Results:**

The analytic cohort comprised 198 patients with a surgical indication: 62 (31.3%) in the In-Time group and 136 (68.7%) in the Delayed group (78 Delayed-operated and 58 Delayed-never-operated). The interval from symptom onset to hospital admission was the largest single contributor to the total time to surgery (∼50% of the total). Crude in-hospital mortality was 9.7% in the In-Time group and 40.4% in the Delayed group (*p* < 0.001). Within the Delayed group, in-hospital mortality was 20.5% in Delayed-operated patients and 67.2% in Delayed-never-operated patients. In the multivariable model (*n* = 193, events = 60), delayed treatment remained independently associated with in-hospital mortality [adjusted OR 3.37, 95% confidence interval (CI) 1.19–9.56; *p* = 0.022]; SOFA score [adjusted odds ratio (aOR) 1.43 per point, 1.23–1.66; *p* < 0.001] and the presence of any embolization (aOR 2.42, 1.09–5.38; *p* = 0.030) were also independently significant.

**Conclusions:**

Delays are common along the IE care pathway and concentrated in the pre-admission and indication-to-surgery phases. Patients with delayed management have substantially higher crude and adjusted in-hospital mortality, with the worst outcomes in patients who never reach the operating room. These observations support modifiable system-level priorities: educating general practitioners and high-risk patients about suspicious symptoms; faster in-hospital access to TOE and PET/CT; and implementing a structured endocarditis Heart Team with protected operating slots.

## Introduction

Infective endocarditis (IE) remains a high-mortality disease despite substantial improvements in microbiology, multimodality imaging, and surgical techniques. Contemporary registries report in-hospital mortality between 17% and 25%, rising to 30% or more in patients with complications such as acute heart failure (AHF), septic shock, or central nervous system embolization (1–3). European and North American guidelines recommend early surgery in patients with valve dysfunction, dehiscence, heart failure, paravalvular abscess, persistent bacteraemia, or high embolic risk and specify time windows for emergency (<24 h), urgent (<5 days), and elective procedures (1, 4). These recommendations rest largely on expert consensus and observational data; the only randomized trial available suggested a benefit of early surgery in patients with severe regurgitation and large vegetations, with no signal of harm from earlier operation (5).

In real-world practice, however, adherence to recommended timing is variable. The EURO-ENDO registry documented that nearly half of patients with a surgical indication did not undergo operation within the recommended interval, and that absence of surgery when indicated was one of the strongest predictors of mortality (6, 7). Delays may originate at several discrete points along the care pathway: patient-related delay in seeking care, primary-care recognition delay, diagnostic delay due to limited access to echocardiography or [18F] FDG-PET/CT, and Heart Team or operative-scheduling delay (8–10). Each step is potentially modifiable, but the relative contribution of these phases has not been systematically quantified in a single cohort.

We addressed this gap by analyzing a consecutive series of definite IE cases collected over 26 years at a regional hospital. Our objectives were (i) to quantify delays at each phase of the IE care pathway in patients with a surgical indication; (ii) to compare in-hospital and 1-year mortality between patients managed within and outside guideline-recommended timing and within prespecified subgroups of the Delayed population; and (iii) to estimate the association between delay and mortality after adjustment for baseline disease severity. We also propose targeted, system-level actions to reduce avoidable delays.

## Methods

### Study design and population

**Figure 1:**
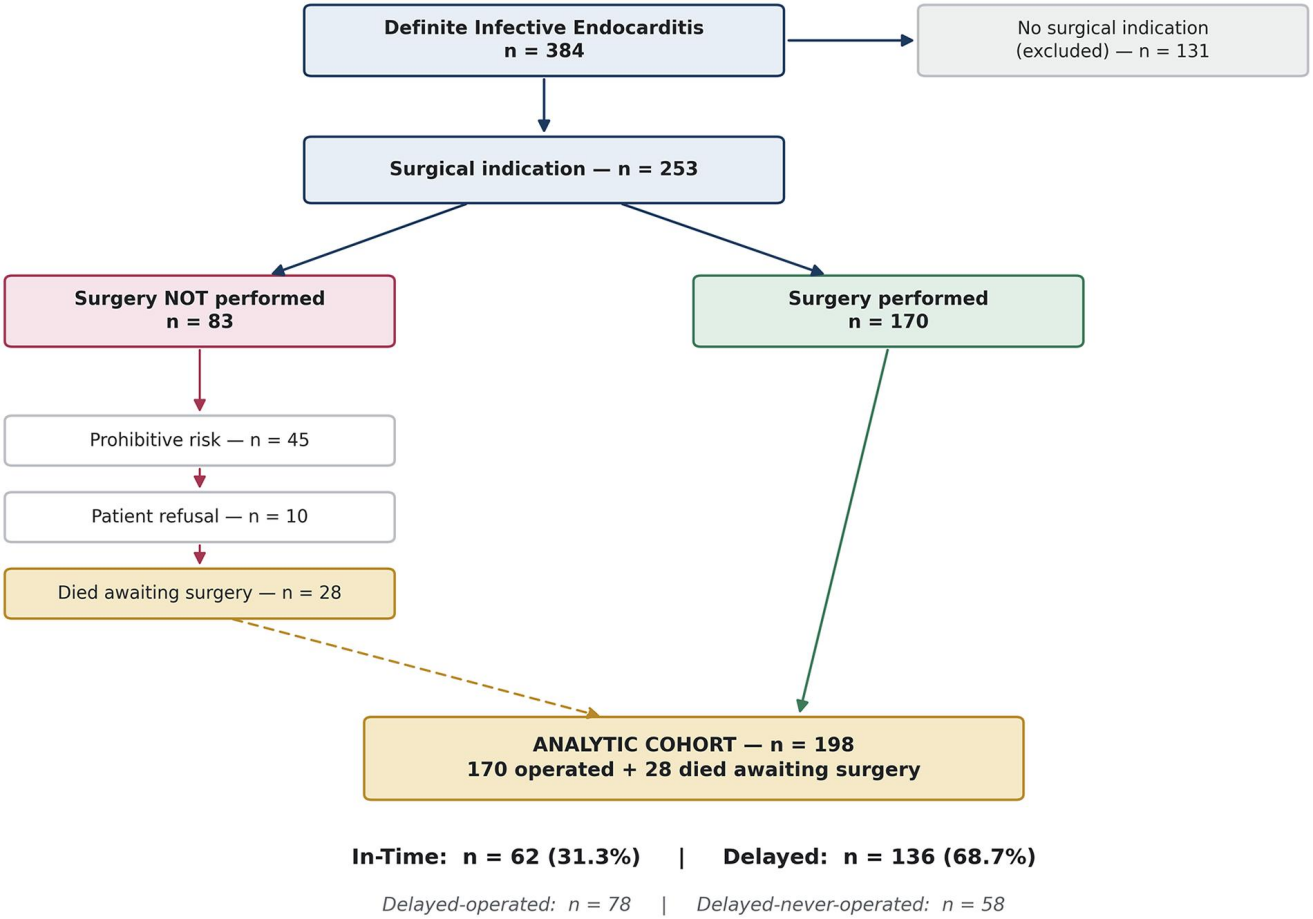
Patient selection flowchart. Of 384 consecutive episodes of definite infective endocarditis diagnosed at our centre between 1 January 1998 and 31 December 2023, 130 patients had no surgical indication and were excluded. Of the remaining 254 patients with a guideline-based surgical indication, the analytic cohort (*n* = 198) was classified consistently into In-Time (*n* = 62; 31.3%) and Delayed (*n* = 136; 68.7%) groups. The Delayed group was further divided into Delayed-operated (*n* = 78) and Delayed-never-operated (*n* = 58).

This is a single-centre, retrospective observational cohort study based on a clinical registry of consecutive patients with suspected IE referred to the Cardiology Department of Misericordia Hospital, Grosseto, Italy. The registry has been maintained prospectively for clinical-quality purposes since 1998; analyses for the present study were performed retrospectively. We included all patients with a definite diagnosis of IE according to the criteria in force at the time of admission (Duke and modified Duke/2023 ESC criteria, as appropriate) (1, 11) who had a guideline-based indication for surgery between 1 January 1998 and 31 December 2023. The selection process is summarized in Figure 1.

Patients were classified into two prespecified groups according to whether the actual time from surgical indication to operation met the timing recommended by current guidelines (emergency within 24 h, urgent within 5 days, and elective otherwise) (1): In-Time (operation within the target) and Delayed (operation outside the target, including patients who never reached the operating room). A prespecified subgroup analysis within the Delayed group distinguished Delayed-operated patients from Delayed-never-operated patients (those with a surgical indication who did not undergo operation because of clinical deterioration, prohibitive risk, refusal, or other reasons).

### Variable definitions

The first symptom was defined as the earliest patient-reported symptom subsequently attributable to IE on retrospective chart review, prioritized in the following hierarchy when more than one symptom was reported: (i) persistent fever (≥38°C for ≥48 h), (ii) embolic event (stroke, peripheral or visceral embolism), (iii) new dyspnoea or signs of heart failure, and (iv) constitutional symptoms (weight loss, asthenia, night sweats) lasting ≥1 week. The date of the first symptom was extracted from the admission history. We acknowledge that the timing of symptom onset is vulnerable to recall bias and that the symptom-to-admission interval reflects a combination of natural disease progression and patient- and system-related delays.

The four prespecified intervals were (i) symptom-to-admission, (ii) admission-to- transthoracic echocardiography (TTE), (iii) TTE-to-transoesophageal echocardiography (TOE), and (iv) TOE-to-surgery. High-risk features included prosthetic valves, previous IE, and intravenous drug abuse (IVDA).

### Data collection

For each patient, we recorded baseline demographics, predisposing conditions, comorbidity burden (Charlson comorbidity index), severity at admission [Sequential Organ Failure Assessment (SOFA) score, NYHA class, haemodynamic and ventilation requirements], microbiological data, and echocardiographic findings. Outcomes were in-hospital all-cause mortality and 1-year all-cause mortality; 1-year follow-up was complete for all included patients.

### Root-cause classification of patients with delayed/no surgery and in-hospital death

For all patients in the Delayed-never-operated subgroup who died in hospital, a structured chart review classified the predominant reason for the absence of surgery into one of the four prespecified categories: (i) clinical deterioration before scheduled surgery (sepsis progression, new neurological event, multi-organ failure); (ii) operating-room or transfer-pathway logistics; (iii) Heart Team re-evaluation due to emerging high-risk features (e.g., cerebral haemorrhage); and (iv) other/unclassifiable.

### Statistical analysis

Continuous variables are presented as mean ± standard deviation (SD) or median (interquartile range) according to the Shapiro–Wilk and Kolmogorov–Smirnov tests for normality. Comparisons of continuous variables between independent groups used the Student *t*-test or the Mann–Whitney *U*-test as appropriate. Categorical variables are presented as *n* (%) and compared with the chi-square or Fisher's exact test.

The association between management timing (In-Time vs. Delayed) and in-hospital mortality was examined using univariable and multivariable logistic regression. Candidate covariates for the multivariable model were prespecified on clinical grounds (age, SOFA score, *Staphylococcus aureus* aetiology, acute heart failure, embolization, paravalvular abscess) and entered as a parsimonious block to respect the events-per-variable rule given the limited number of events; no automated variable selection was applied. Effect sizes are reported as odds ratios (ORs) with 95% confidence intervals (CIs). Kaplan–Meier curves for in-hospital (90-day follow-up) and 1-year survival are presented and compared using the log-rank test. A two-sided *p*-value <0.05 was considered statistically significant. Analyses were performed in R version 4.4 (R Core Team, Vienna) using the survival and rms packages.

## Results

### Cohort and baseline characteristics

Between 1 January 1998 and 31 December 2023, 384 consecutive episodes of definite IE were diagnosed at our centre. The annual hospital admission rate for IE during the study period was approximately 15 cases/year. We do not present a province-level incidence estimate because our institution does not capture every IE case in the catchment area. A surgical indication according to contemporary guidelines was present in 253 patients (65.9%); the analytic cohort, after applying the consistent classification described in the Methods section, comprised 198 patients (Figure 1).

Baseline characteristics are reported in Table 1. The In-Time group included 62 patients (31.3%) and the Delayed group included 136 patients (68.7%); within the Delayed group, 78 patients (57.4%) underwent surgery outside the recommended window (Delayed-operated), whereas 58 (42.6%) did not undergo surgery (Delayed-never-operated). Age and sex distribution did not differ significantly between groups; the Delayed group had numerically higher SOFA scores and a higher proportion of patients with NYHA class > II at presentation.

**Table 1:** Baseline characteristics of the In-Time and Delayed groups.

Characteristic	Total (*n* = 198)	In-Time (*n* = 62)	Delayed (*n* = 136)	*p*-Value
Male, *n* (%)	118 (59.6)	30 (48.4)	88 (64.7)	0.03
Age, years (mean ± SD)	62.7 ± 16.5	61.8 ± 16.6	63.5 ± 16.3	0.25
ICU admission, *n* (%)	73 (36.9)	23 (37.1)	50 (36.8)	0.96
Orotracheal intubation, *n* (%)	23 (11.6)	7 (11.3)	16 (11.8)	0.92
Comorbidities				
Active cancer, *n* (%)	12 (6.1)	4 (6.5)	8 (5.9)	0.87
Diabetes mellitus, *n* (%)	35 (17.7)	12 (19.4)	23 (16.9)	0.67
Ischaemic heart disease, *n* (%)	25 (12.6)	11 (17.7)	14 (10.3)	0.14
COPD, *n* (%)	24 (12.1)	8 (12.9)	16 (11.8)	0.82
Peripheral arterial disease, *n* (%)	27 (13.6)	18 (29.0)	9 (6.6)	<0.001
Previous stroke, *n* (%)	29 (14.6)	9 (14.5)	20 (14.7)	0.97
History of heart failure, *n* (%)	62 (31.3)	25 (40.3)	37 (27.2)	0.06
Atrial fibrillation, *n* (%)	31 (15.7)	10 (16.1)	21 (15.4)	0.90
Immunocompromised, *n* (%)	10 (5.1)	3 (4.8)	7 (5.1)	0.93
Clinical severity at admission				
LVEF (%), mean ± SD	58.2 ± 8.4	58.5 ± 8.5	58.0 ± 8.1	0.54
NYHA class > II, *n* (%)	67 (33.8)	19 (30.6)	48 (35.3)	0.52
SOFA score, mean ± SD	3.1 ± 2.8	2.7 ± 2.6	3.5 ± 3.0	0.07
Charlson comorbidity index, mean ± SD	2.4 ± 1.8	2.2 ± 1.9	2.6 ± 1.8	0.18
Creatinine (mg/dL), mean ± SD	1.75 ± 1.45	1.60 ± 1.18	1.81 ± 1.71	0.16
Dialysis, *n* (%)	6 (3.0)	3 (4.8)	3 (2.2)	0.31

Continuous variables as mean ± SD; categorical as *n* (%). *P*-values were from the chi-square/Fisher (categorical) or *t*-test/Mann–Whitney *U*-test (continuous). COPD, chronic obstructive pulmonary disease; ICU, intensive care unit; LVEF, left ventricular ejection fraction; NYHA, New York Heart Association; SOFA, Sequential Organ Failure Assessment.

### Clinical and microbiological features

**Figure 2:**
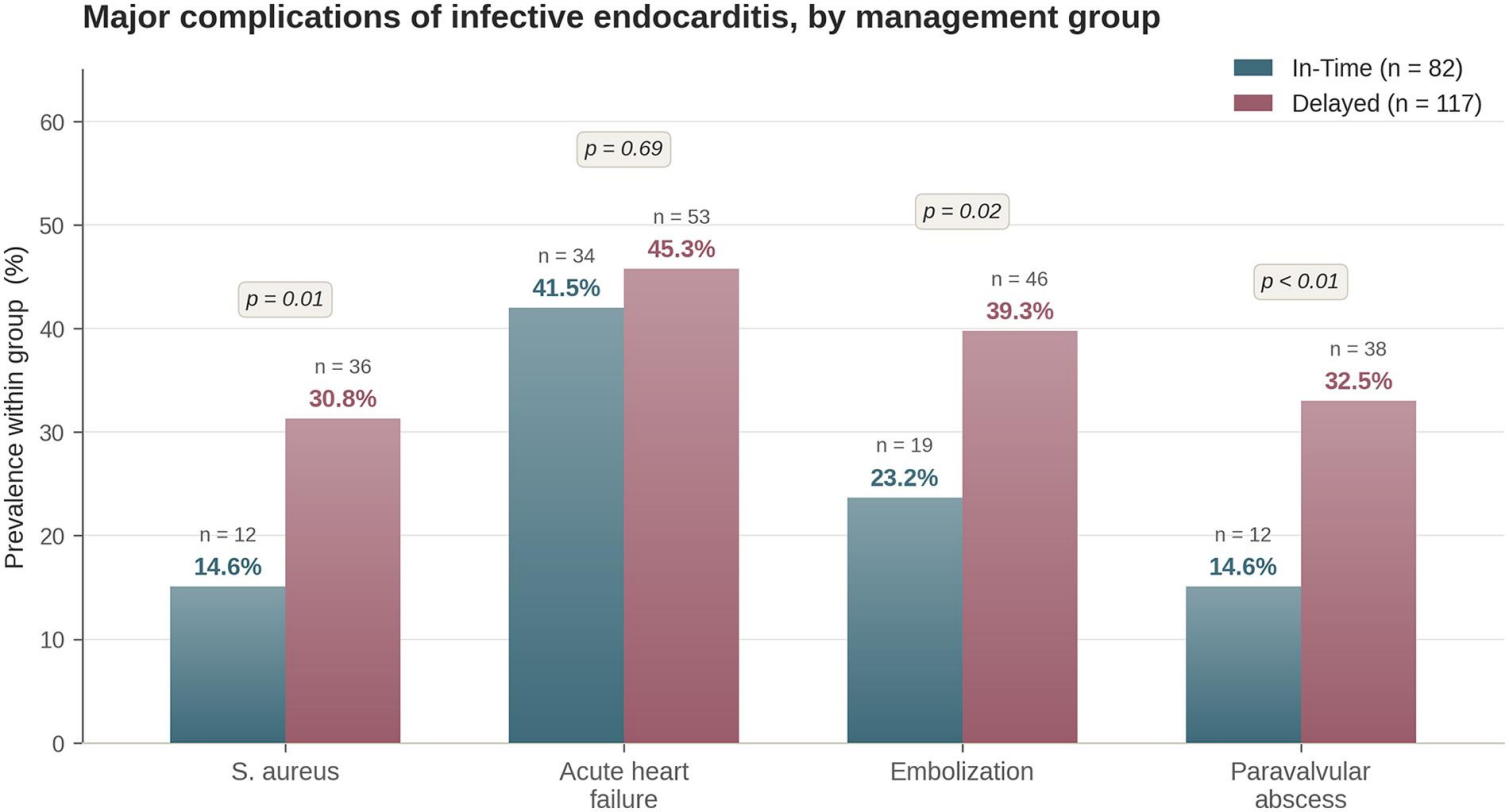
Major complications of infective endocarditis, by the management group. Prevalence (within each group) of *S. aureus* aetiology, acute heart failure (AHF), any embolization, and paravalvular abscess in the In-Time (*n* = 62) and Delayed (*n* = 136) groups. Values above each bar are the proportion (%) and absolute count. *P*-values from the chi-square or Fisher's exact test.

The aortic valve was the most frequently affected (37.7%), followed by the mitral valve (36.7%), the tricuspid valve (7.5%), and the pulmonary valve (0.5%); five patients had multivalvular involvement. *S. aureus* was the most frequently isolated pathogen (24.1%), followed by coagulase-negative staphylococci (13.6%), the *Streptococcus viridans* group (11.6%), and enterococci (4.5%); blood cultures were negative in 14.6% of patients. Compared with the In-Time group, patients in the Delayed group more frequently presented with embolization, paravalvular abscess, AHF, and *S. aureus* infection (all *p* < 0.05) (Table 2, Figure 2).

**Table 2:** Clinical, microbiological, and outcome features in the In-Time and Delayed groups.

Feature	Total (*n* = 198)	In-Time (*n* = 62)	Delayed (*n* = 136)	*p*-Value
High-risk IE features, *n* (%)	84 (42.4)	53 (85.5)	31 (22.8)	<0.001
Intravenous drug abuse, *n* (%)	23 (11.6)	9 (14.5)	14 (10.3)	0.39
Prosthetic valves, *n* (%)	61 (30.8)	20 (32.3)	41 (30.1)	0.76
Previous IE, *n* (%)	5 (2.5)	1 (1.6)	4 (2.9)	0.60
Affected valve				
Aortic, *n* (%)	75 (37.9)	25 (40.3)	50 (36.8)	0.63
Mitral, *n* (%)	73 (36.9)	23 (37.1)	50 (36.8)	0.97
Tricuspid, *n* (%)	15 (7.6)	5 (8.1)	10 (7.4)	0.86
CDRIE, *n* (%)	13 (6.6)	2 (3.2)	11 (8.1)	0.19
Complications				
Acute heart failure, *n* (%)	87 (43.9)	26 (41.9)	61 (44.9)	0.70
Embolization (any), *n* (%)	65 (32.8)	14 (22.6)	51 (37.5)	0.04
Cerebral, *n* (%)	36 (18.2)	8 (12.9)	28 (20.6)	0.19
Paravalvular abscess, *n* (%)	50 (25.3)	9 (14.5)	41 (30.1)	0.02
Microbiology				
Negative blood cultures, *n* (%)	29 (14.6)	8 (12.9)	21 (15.4)	0.63
*S. aureus*, *n* (%)	48 (24.2)	9 (14.5)	39 (28.7)	0.03
*S. viridans* group, *n* (%)	23 (11.6)	8 (12.9)	15 (11.0)	0.69
Coagulase-negative staphylococci, *n* (%)	27 (13.6)	6 (9.7)	21 (15.4)	0.26
Enterococci, *n* (%)	9 (4.5)	3 (4.8)	6 (4.4)	0.89
Surgical indication				
Urgent, *n* (%)	162 (81.8)	39 (62.9)	123 (90.5)	<0.001
Emergency, *n* (%)	12 (6.1)	3 (4.8)	9 (6.6)	0.92
Elective, *n* (%)	24 (12.1)	20 (32.3)	4 (2.9)	<0.001
Outcomes				
In-hospital death, *n* (%)	61 (30.8)	6 (9.7)	55 (40.4)	<0.001
1-year all-cause death, *n* (%)	70 (35.4)	7 (11.3)	63 (46.3)	<0.001

CDRIE, cardiac-device-related infective endocarditis; IE, infective endocarditis; IVDA, intravenous drug abuse.

### Time intervals along the care pathway

**Figure 3:**
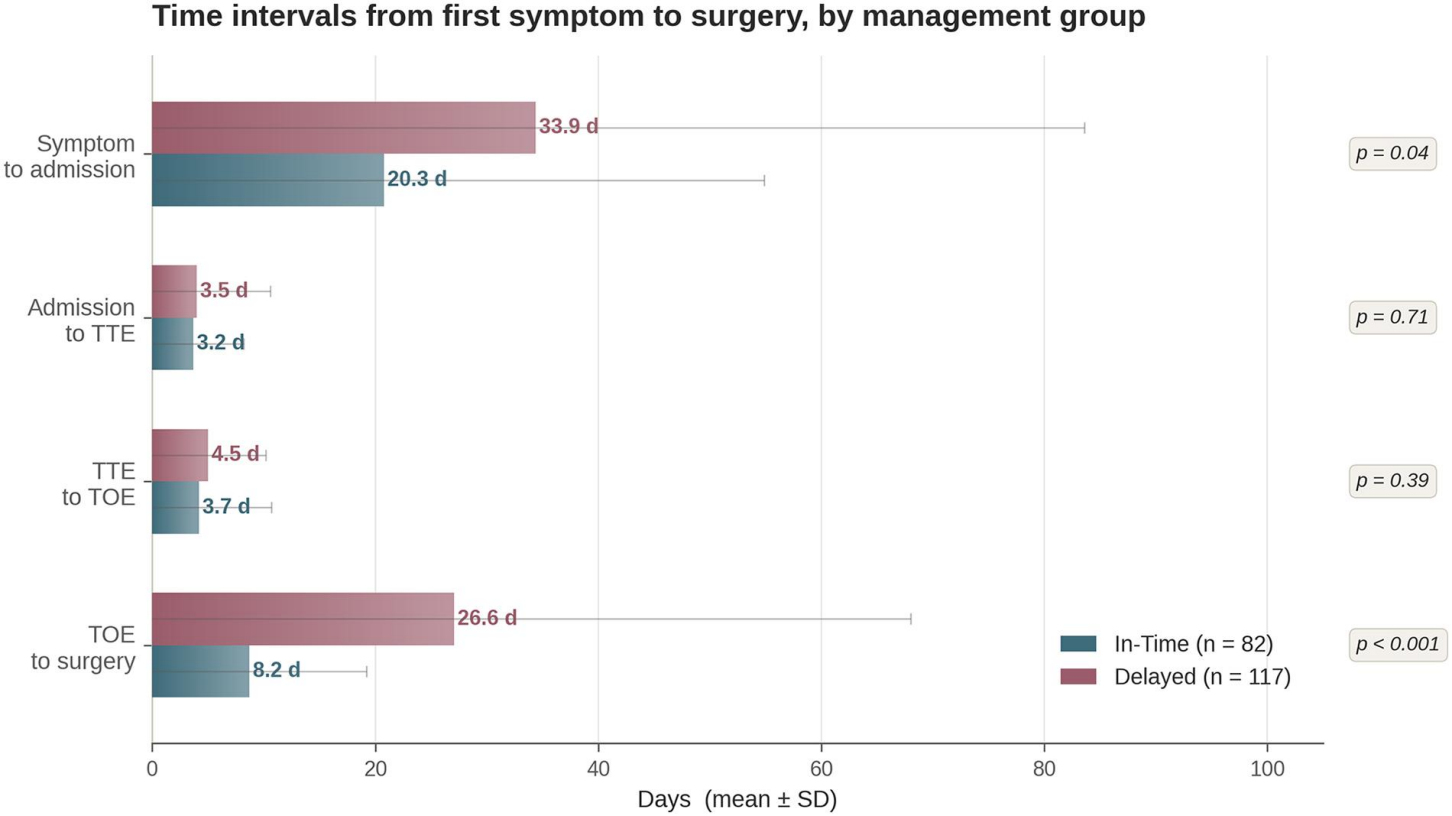
Time intervals from the first symptom to surgery, by the management group. Mean ± SD (days) for the four pre-specified intervals along the IE care pathway. The symptom-to-admission and TOE-to-surgery intervals account for the majority of the total time-to-surgery and differ significantly between groups. Comparisons used the Mann–Whitney *U*-test. TTE, transthoracic echocardiography; TOE, transoesophageal echocardiography.

The symptom-to-admission interval was the dominant component of total time-to-surgery in both groups (mean 20.3 ± 34.6 days in the In-Time group vs. 33.9 ± 49.7 days in the Delayed group, *p* = 0.04), accounting for approximately 50% of the cumulative interval. The admission-to-TTE and TTE-to-TOE intervals were short and similar between groups. The TOE-to-surgery interval was 8.2 ± 11.0 days in the In-Time group and 26.6 ± 41.4 days in the Delayed group (*p* < 0.001) (Table 3, Figure 3).

**Table 3:** Time intervals along the IE care pathway, by the management group.

Interval	Group	*N*	Mean ± SD (days)	*p*-Value
Symptom to admission	In-Time	62	20.3 ± 34.6	0.04
	Delayed	136	33.9 ± 49.7	
Admission to TTE	In-Time	62	3.2 ± 5.0	0.71
	Delayed	136	3.5 ± 7.1	
TTE to TOE	In-Time	62	3.7 ± 7.0	0.39
	Delayed	136	4.5 ± 5.7	
TOE to surgery	In-Time	62	8.2 ± 11.0	<0.001
	Delayed	136	26.6 ± 41.4	

Mann–Whitney *U*-test. TTE, transthoracic echocardiography; TOE, transoesophageal echocardiography.

### Diagnostic yields of TTE and TOE

**Figure 4:**
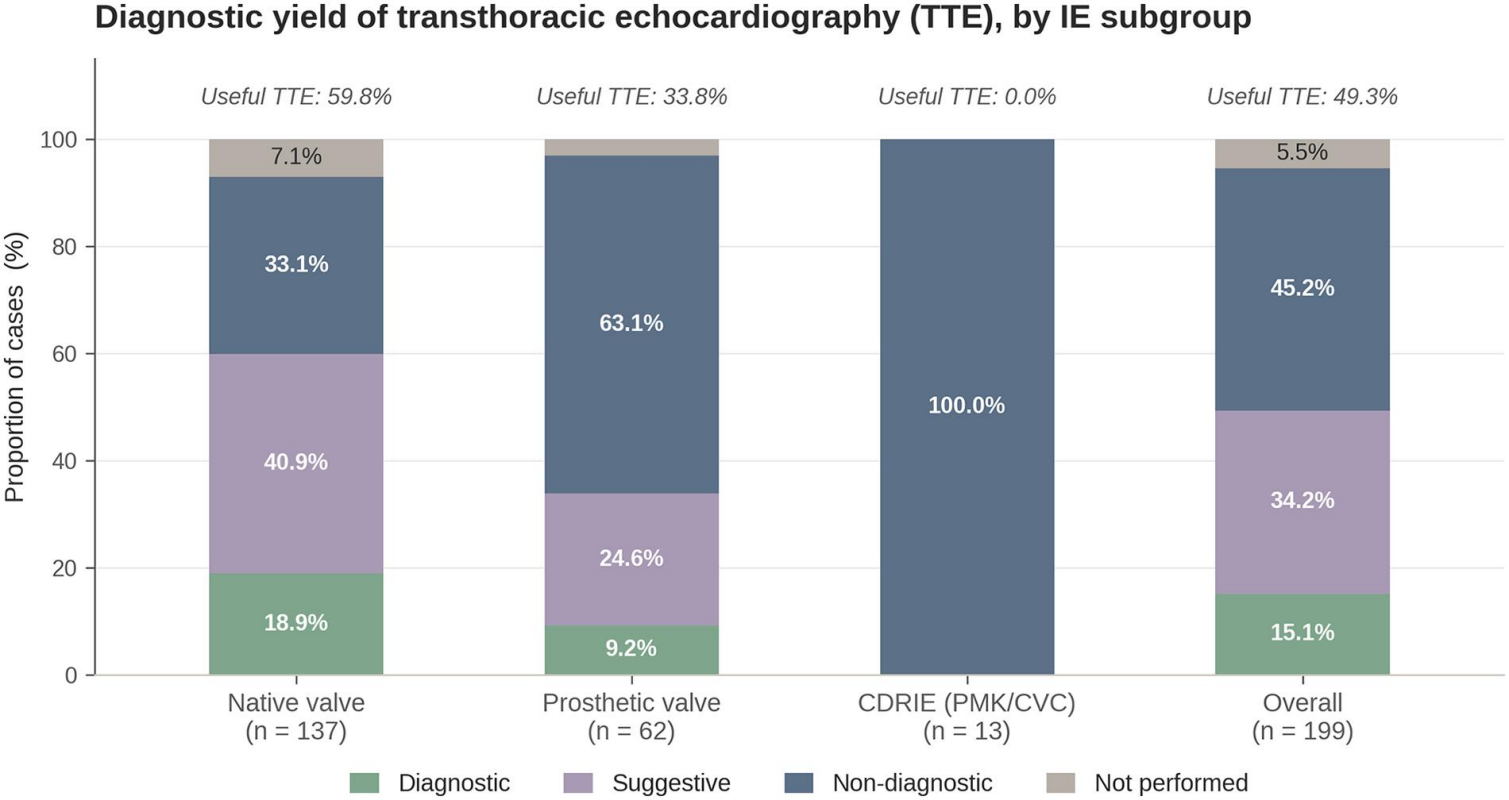
Diagnostic yield of transthoracic echocardiography (TTE) alone, by the IE subgroup. Each bar shows the distribution of TTE findings (diagnostic, suggestive, non-diagnostic, not performed) within four IE subgroups: native-valve IE (*n* = 137), prosthetic-valve IE (*n* = 62), cardiac-device-related IE (CDRIE, PMK/CVC) (*n* = 13), and the overall cohort (*n* = 199). The combined proportion of Diagnostic and Suggestive findings (“Useful TTE”) is annotated above each bar: 59.8% in native-valve IE, 33.8% in prosthetic-valve IE, 0% in CDRIE, and 49.3% overall. TTE was diagnostic alone in 18.9% of native-valve IE, 9.2% of prosthetic-valve IE, and 0% of CDRIE, confirming the lower TTE yield in the presence of prosthetic materials or intracardiac devices. CDRIE, cardiac-device-related infective endocarditis; CVC, central venous catheter; IE, infective endocarditis; PMK, pacemaker; TTE, transthoracic echocardiography.

As displayed in Figure 4, the diagnostic yield of TTE alone varied markedly by clinical context: TTE was diagnostic in 18.9% of native-valve IE, 9.2% of prosthetic-valve IE, and 0% of cardiac-device-related IE (CDRIE) involving a pacemaker or central-venous-catheter leads. When combining Diagnostic and Suggestive findings (“useful TTE”), the proportion was 59.8% in native-valve IE, 33.8% in prosthetic-valve IE, and 0% in CDRIE, confirming the lower TTE yield in the presence of prosthetic material or intracardiac devices.

### Mortality and association with delay

**Figure 5:**
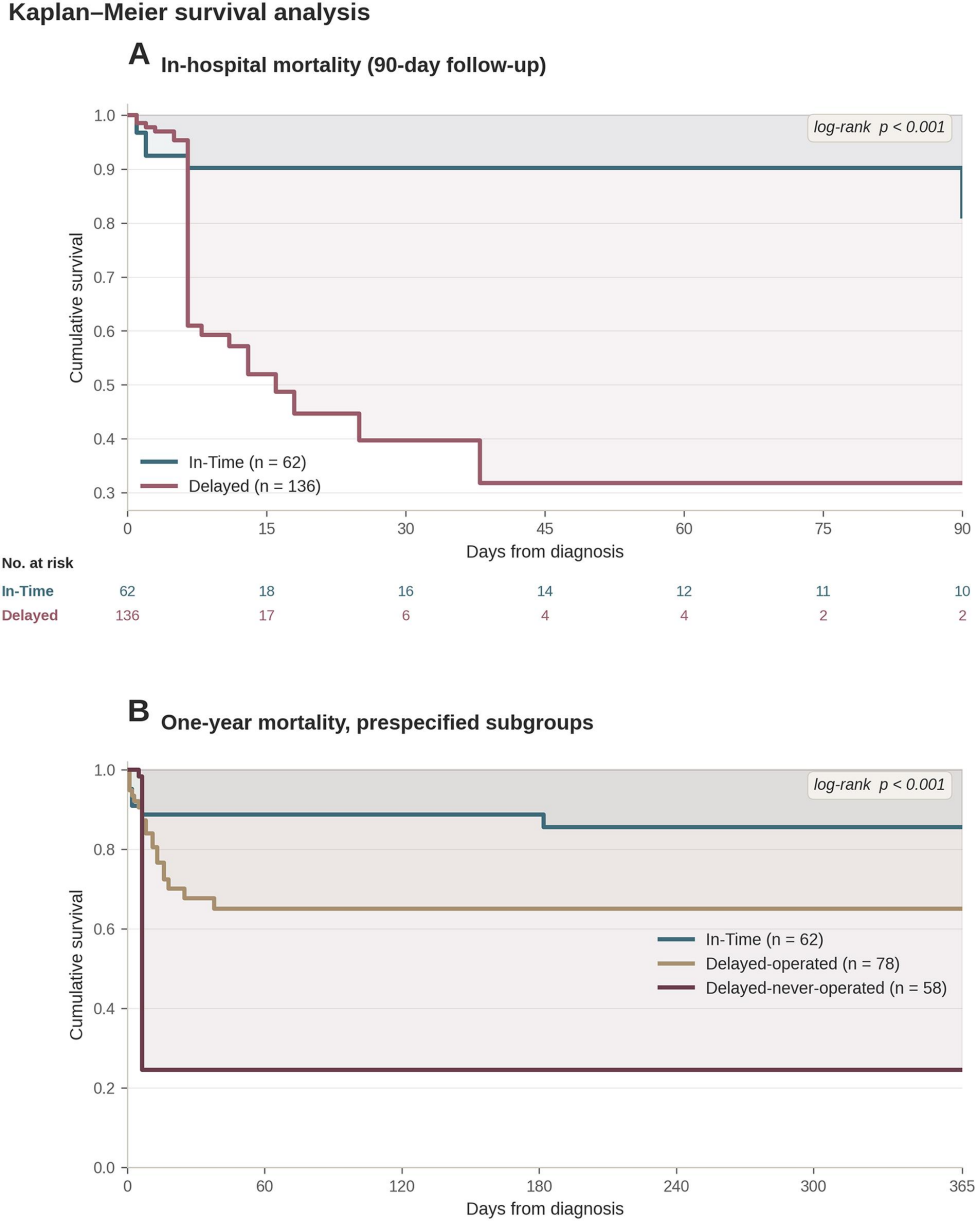
Kaplan–Meier survival analysis. **(A)** In-hospital mortality (90-day follow-up) in the In-Time (*n* = 62, blue) and Delayed (*n* = 136, plum) groups. At-risk numbers are shown below the panel. **(B)** One-year mortality in the pres-pecified subgroups: In-Time (*n* = 62, blue), Delayed-operated (*n* = 78, taupe), and Delayed-never-operated (*n* = 58, dark plum). Comparisons used the log-rank test. The figure illustrates that most of the mortality excess in the Delayed group is concentrated in the Delayed-never-operated subgroup, i.e., in patients who never reached the operating room.

In-hospital mortality occurred in 61 of 198 patients (30.8%), and 1-year mortality occurred in 70 patients (35.4%). Crude in-hospital mortality was 6/62 (9.7%) in the In-Time group and 55/136 (40.4%) in the Delayed group (*p* < 0.001). Within the Delayed group, the prespecified subgroup analysis showed a striking gradient: Delayed-operated patients had an in-hospital mortality of 16/78 (20.5%), whereas Delayed-never-operated patients had an in-hospital mortality of 39/58 (67.2%). One-year mortality mirrored the in-hospital pattern (Table 4, Figure 5).

**Table 4:** Crude in-hospital and 1-year mortality in the pre-specified subgroups.

Subgroup	*N*	In-hospital mortality, *n* (%)	1-year mortality, *n* (%)
In-Time	62	6 (9.7)	7 (11.3)
Delayed-operated	78	16 (20.5)	20 (25.6)
Delayed-never-operated	58	39 (67.2)	43 (74.1)
Overall analytic cohort	198	61 (30.8)	70 (35.4)

Subgroups are pre-specified. The Delayed-never-operated subgroup includes patients with a guideline-based surgical indication who did not undergo operation for any reason (clinical deterioration/death before surgery, prohibitive surgical risk, patient refusal). One-year follow-up was complete for all included patients.

**Figure 6:**
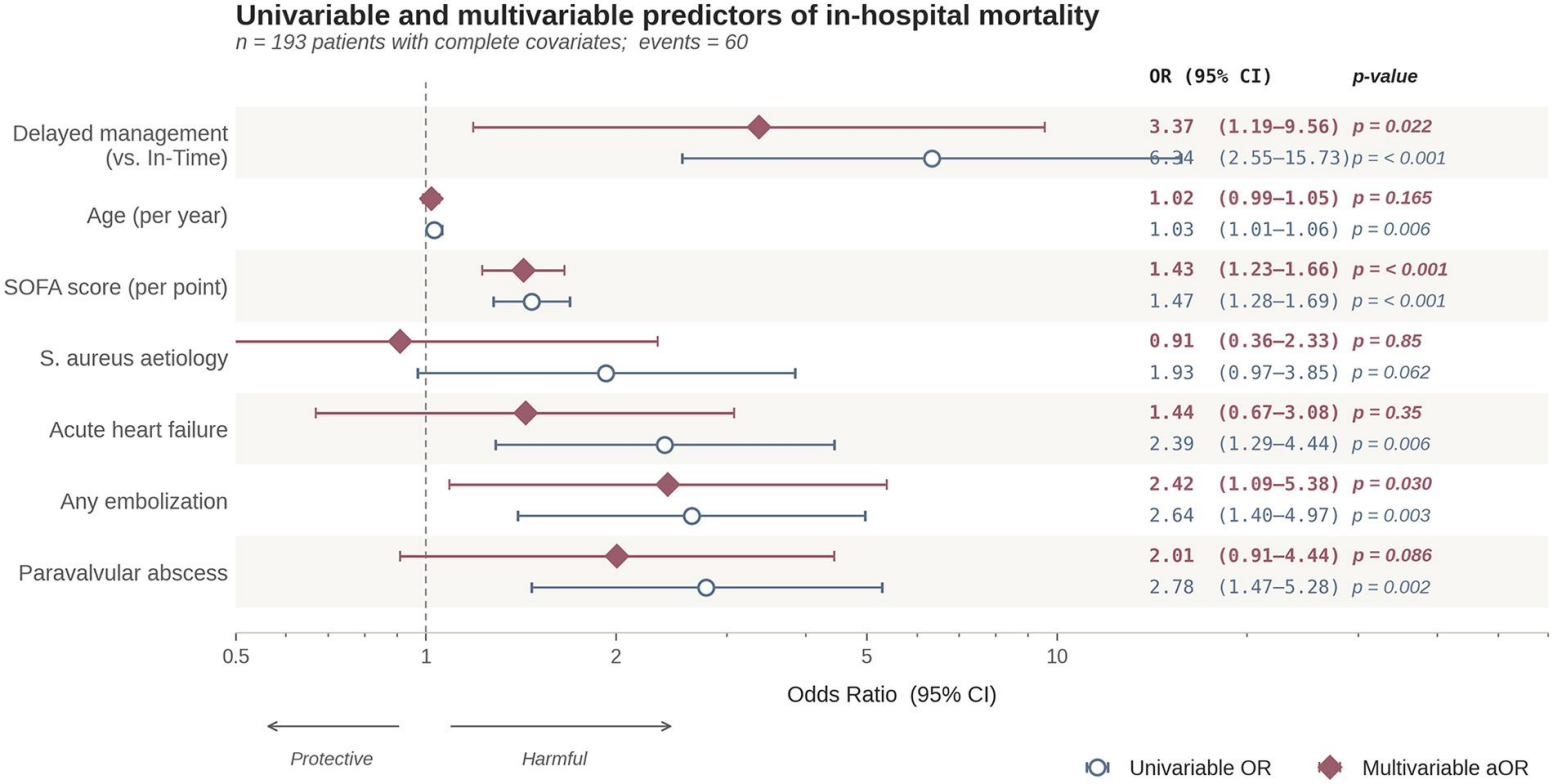
Univariable and multivariable predictors of in-hospital mortality. Forest plot of odds ratios (ORs) with 95% confidence intervals for the association between candidate predictors and in-hospital mortality. Univariable estimates are shown in slate circles; multivariable adjusted estimates from the pre-specified parsimonious model are shown in plum diamonds. Numeric values and *p*-values are reported to the right of each row. The multivariable model was fitted in *n* = 193 patients with complete covariate data (events = 60). After adjustment, delay remains independently associated with in-hospital mortality (aOR 3.37, 95% CI 1.19–9.56; *p* = 0.022); SOFA score (per point) and any embolization are the other independently significant predictors. aOR, adjusted odds ratio; CI, confidence interval; OR, odds ratio; SOFA, Sequential Organ Failure Assessment.

In univariable analysis, delay (OR 6.34, 95% CI 2.55–15.73; *p* < 0.001), age, SOFA score, AHF, embolization, and abscess were each associated with in-hospital mortality, and *S. aureus* showed borderline significance (Figure 6). In the prespecified multivariable logistic-regression model (*n* = 193 with complete covariates, events = 60), delay remained independently associated with in-hospital mortality after adjustment for age, SOFA, *S. aureus*, AHF, embolization, and abscess (adjusted OR 3.37, 95% CI 1.19–9.56; *p* = 0.022). SOFA score [adjusted odds ratio (aOR) 1.43 per point, 95% CI 1.23–1.66; *p* < 0.001] and any embolization (aOR 2.42, 95% CI 1.09–5.38; *p* = 0.030) were also independently significant predictors; paravalvular abscess showed a borderline association (aOR 2.01, 95% CI 0.91–4.44; *p* = 0.086) (Table 5). These findings indicate that the excess mortality observed in the Delayed group is only partly explained by baseline severity: a substantial independent component remains attributable to the delay itself or, more accurately, to the upstream factors that cause it.

**Table 5:** Univariable and multivariable logistic regression for in-hospital mortality.

Variable	Univariable OR (95% CI)	Univariable *p*	Multivariable aOR (95% CI)	Multivariable *p*
Delayed management (vs. In-Time)	6.34 (2.55–15.73)	<0.001	3.37 (1.19–9.56)	0.022
Age (per year)	1.03 (1.01–1.06)	0.006	1.02 (0.99–1.05)	0.165
SOFA score (per point)	1.47 (1.28–1.69)	<0.001	1.43 (1.23–1.66)	<0.001
*S. aureus* aetiology	1.93 (0.97–3.85)	0.062	0.91 (0.36–2.33)	0.85
Acute heart failure at presentation	2.39 (1.29–4.44)	0.006	1.44 (0.67–3.08)	0.35
Any embolization	2.64 (1.40–4.97)	0.003	2.42 (1.09–5.38)	0.030
Paravalvular abscess	2.78 (1.47–5.28)	0.002	2.01 (0.91–4.44)	0.086

Multivariable model fitted in *N* = 193 patients with complete covariate data (events = 60). Pre-specified parsimonious model; no automated variable selection. aOR, adjusted odds ratio; CI, confidence interval; OR, odds ratio; SOFA, Sequential Organ Failure Assessment.

## Discussion

In a consecutive 26-year cohort of patients with definite IE and a guideline-based indication for surgery, we found that (i) the symptom-to-admission interval was the largest single contributor to total time-to-surgery; (ii) patients managed outside recommended timing had substantially higher crude in-hospital and 1-year mortality; (iii) delay remained independently associated with in-hospital mortality after adjustment for age, SOFA score, microbiology, AHF, embolization, and abscess; and (iv) the highest mortality was concentrated in the Delayed-never-operated subgroup, in which two-thirds of patients died in hospital. These findings extend the EURO-ENDO observation that failure to operate when indicated is among the strongest predictors of mortality and suggest that a portion of the prognostic effect of delay is not fully captured by conventional severity covariates.

### Where the delays occur, and how to reduce them

#### Symptom-to-admission

This interval—20 days in the In-Time and 34 days in the Delayed group—accounted for approximately half of the total time-to-surgery. It reflects a mixture of patient-dependent factors and primary-care recognition and cannot be assumed to be entirely modifiable. Reasonable system-level actions include structured education for general practitioners on persistent fever in patients with prosthetic material, recent invasive procedures, or known valvular disease (8); systematic blood-culture collection for patients with fever of unknown origin in these populations (12); and patient-facing information for known high-risk individuals.

#### Admission-to-TTE

This interval was short (≈3 days in both groups) and unlikely to drive outcomes in our cohort, although emergency-department point-of-care echocardiography in patients with unexplained fever and a predisposing cardiac substrate is a low-cost step that could compress it further.

#### TTE-to-TOE

The mean 4-day TTE-to-TOE interval is potentially modifiable. TOE should be performed without delay whenever IE is suspected, given the limited diagnostic yield of TTE in prosthetic-valve, intracardiac-device, and central-venous-catheter IE (Figure 4). We have previously shown that mitral–aortic intervalvular fibrosa thickening >7 mm on TOE has diagnostic value comparable to that of overt abscess and is sufficient to indicate urgent surgery without waiting for typical vacuolization (13). [18F]FDG-PET/CT can help clarify dubious cases involving prosthetic material (9).

#### Indication-to-surgery

This interval differed most strongly between groups (8 vs. 27 days) and is the most directly actionable. Cerebral septic embolism—the most feared driver of surgical postponement—is no longer a contraindication to early operation in most circumstances; current guidelines explicitly state that surgery should not be delayed after an ischaemic stroke and after intracranial haemorrhage individualized decision-making by the Heart Team is recommended (1, 14). In our cohort, the Delayed-never-operated subgroup accounted for the majority of deaths and represents the principal target for system-level intervention: formalized endocarditis team protocols, dedicated operating slots, and pre-existing transfer agreements with hub centres.

### Comparison with previous literature

Our findings are consistent with the EURO-ENDO registry, in which failure to operate when indicated emerged as the most powerful single predictor of mortality, and with subsequent EURO-ENDO subanalyses in left-sided IE complicated by heart failure (6, 7). We extend this evidence by decomposing the time-to-surgery into discrete intervals and by formally separating, within the Delayed group, the prognostic contribution of delayed operation vs. no operation. Importantly, our multivariable analysis suggests that a portion of the prognostic effect of delay persists after adjustment for clinical severity, consistent with the hypothesis that delay itself contributes causally, although residual confounding cannot be excluded in observational data.

### Limitations

Our study has several important limitations. First, it is a single-centre observational cohort spanning a long period (26 years), during which diagnostic criteria, imaging technology, microbiological techniques, referral pathways, and intensive-care practice all evolved; this temporal heterogeneity cannot be fully captured by simple covariate adjustment. Second, confounding by indication and baseline severity is intrinsic to comparisons of timing in observational data, and our multivariable models can only partially address this. Third, the symptom-to-admission interval is vulnerable to recall bias, and the definition of the “first symptom” inherently mixes specific (embolic, heart-failure) and non-specific (constitutional) presentations; the operational definition we adopted is reasonable but not a gold standard. Fourth, the Delayed-never-operated subgroup is a heterogeneous category that includes patients who died before reachable surgery, patients with prohibitive surgical risk in whom the Heart Team declined operation, and patients who refused surgery; further sub-classification is reported descriptively in the Results section, but the modest number of events limits inferential power. Fifth, the number of events constrains the complexity of the multivariable model; the 95% confidence interval around the adjusted effect of delay is correspondingly wide. Sixth, we did not formally analyse the role of negative blood cultures or delayed [18F]FDG-PET/CT as separate contributors to diagnostic delay; these questions warrant dedicated analyses.

## Conclusions

Delays along the IE care pathway are common and concentrated in the pre-admission and indication-to-surgery phases. Patients managed outside guideline-recommended timing have substantially higher crude and adjusted mortality, with the worst outcomes concentrated in patients who never reach the operating room. These observations support several modifiable system-level priorities: education of general practitioners and high-risk patients on suspicious symptoms, faster in-hospital access to TOE and [18F]FDG-PET/CT, and a formal endocarditis Heart Team with protected operating slots and pre-arranged transfer pathways to cardiac surgery hubs. Although our multivariable analysis suggests an independent effect of delay on mortality, causal claims about the magnitude of mortality reduction achievable by reducing delay should await prospective, multicentre, severity-adjusted analyses.

## Data Availability

The raw data supporting the conclusions of this article will be made available by the authors, without undue reservation.
